# Impacts of Multiwalled Carbon Nanotubes on Nutrient Removal from Wastewater and Bacterial Community Structure in Activated Sludge

**DOI:** 10.1371/journal.pone.0107345

**Published:** 2014-09-19

**Authors:** Reti Hai, Yulin Wang, Xiaohui Wang, Zhize Du, Yuan Li

**Affiliations:** Beijing Engineering Research Center of Environmental Material for Water Purification, Beijing University of Chemical Technology, Beijing, China; University of California, Merced, United States of America

## Abstract

**Background:**

The increasing use of multiwalled carbon nanotubes (MWCNTs) will inevitably lead to the exposure of wastewater treatment facilities. However, knowledge of the impacts of MWCNTs on wastewater nutrient removal and bacterial community structure in the activated sludge process is sparse.

**Aims:**

To investigate the effects of MWCNTs on wastewater nutrient removal, and bacterial community structure in activated sludge.

**Methods:**

Three triplicate sequencing batch reactors (SBR) were exposed to wastewater which contained 0, 1, and 20 mg/L MWCNTs. MiSeq sequencing was used to investigate the bacterial community structures in activated sludge samples which were exposed to different concentrations of MWCNTs.

**Results:**

Exposure to 1 and 20 mg/L MWCNTs had no acute (1 day) impact on nutrient removal from wastewater. After long-term (180 days) exposure to 1 mg/L MWCNTs, the average total nitrogen (TN) removal efficiency was not significantly affected. TN removal efficiency decreased from 84.0% to 71.9% after long-term effects of 20 mg/L MWCNTs. After long-term exposure to 1 and 20 mg/L MWCNTs, the total phosphorus removal efficiencies decreased from 96.8% to 52.3% and from 98.2% to 34.0% respectively. Further study revealed that long-term exposure to 20 mg/L MWCNTs inhibited activities of ammonia monooxygenase and nitrite oxidoreductase. Long-term exposure to 1 and 20 mg/L MWCNTs both inhibited activities of exopolyphosphatase and polyphosphate kinase. MiSeq sequencing data indicated that 20 mg/L MWCNTs significantly decreased the diversity of bacterial community in activated sludge. Long-term exposure to 1 and 20 mg/L MWCNTs differentially decreased the abundance of nitrifying bacteria, especially ammonia-oxidizing bacteria. The abundance of PAOs was decreased after long-term exposure to 20 mg/L MWCNTs. The abundance of glycogen accumulating organisms (GAOs) was increased after long-term exposure to 1 mg/L MWCNTs.

**Conclusion:**

MWCNTs have adverse effects on biological wastewater nutrient removal, and altered the diversity and structure of bacterial community in activated sludge.

## Introduction

Nanomaterials are defined as particles, fibers, and tubes with at least one dimension of 100 nm or less [1]. Nanoparticles have unique chemical and physical properties that allow for their application in a variety of fields; they are used as catalysts, skin creams, semiconductors, and pigments in biomedicine, textiles, cosmetics, and other industries [2], [3]. Among them, carbon nanotubes (CNTs), which are tubular graphite sheets consisting of sp^2^ carbon bonds typically with diameters of 1.4 nm and lengths in the microns, are considered to be novel materials [4]. CNTs have a length-to-diameter ratio significantly larger than any other material and provide a very large surface area per unit weight, and this makes them suitable for applications in high-capacity drug delivery [5]. In addition, metal decorated CNTs have unique properties and are gaining extensive attention for their potential applications as catalysts, broad-band optical limiters, electrodes, and advanced materials [6]. The increasing use of CNTs will lead to an increase in their release to wastewater treatment plants (WWTPs) which play a significant role in preventing potentially hazardous materials from spreading in the environment. It is essential to investigate the possible adverse effects of CNT on WWTPs.

The impacts of metal nanoparticles and metal oxide nanoparticles, such as silver nanoparticles (Ag NPs) [7],[8], copper nanoparticles (Cu NPs) [9], gold nanoparticles (Au NPs) [10], titanium dioxide (TiO_2_) [11,[12], aluminum oxide (Al_2_O_3_) [11], silicon dioxide (SiO_2_) [13], and zinc oxide (ZnO) [14]–[16], on WWTPs have been comprehensively studied. However, few studies have focused on the effects of CNTs on WWTPs. Furthermore, current knowledge of the toxicity of CNTs mainly comes from the study of only certain microorganisms, or under pure culture conditions. For example, by studying the cytotoxicity of CNTs of different sizes (diameters) on *Escherichia coli*, researchers demonstrated that single-wall carbon nanotubes (SWCNTs) are much more toxic to bacteria than multiwalled carbon nanotubes (MWCNTs) [17]. Some other researchers [18] also demonstrated that SWCNTs are more toxic to bacterial pathogens. Higher toxicity in bacterial culture was also observed when the length of MWCNTs was shorter [19]. However, to date, only a very limited number of studies have investigated the effects of CNTs on complex microbial systems. By studying the performance of an activated sludge reactor after a shock exposure to SWCNTs (270 mg/L), Yin et al. [20],[21] found that the addition of SWCNTs did not negatively influence the performance of a continuous reactor; in fact, SWCNTs improved sludge settleability and sludge dewaterability; a decrease of chemical oxygen demand (COD) in effluent was also detected as a result of absorption by SWCNTs. The research of Goyal [22] showed that SWCNTs differentially impacted members of the activated sludge reactor bacterial community. To date, however, no research has been done on the long-term effects of MWCNTs on biological wastewater treatment systems or their bacterial communities.

The activated sludge process is the most widely used biological process to treat municipal and industrial wastewater. The efficient and stable operation of biological WWTPs relies upon the diversity of microbial populations and the stable microbial community structures within them. However it is still unknown whether long-term exposure of MWCNTs affects bacterial communities in activated sludge.

The aim of this study was to investigate the impacts of MWCNTs on nitrogen and phosphorus removal from wastewater and on bacterial community structure and diversity in activated sludge. The short- and long-term effects of MWCNTs on nutrient removal from wastewater and the effects of MWCNTs on the biological activity of activated sludge was studied using activated sludge respiration inhibition test. In addition, the activity of some key enzymes, such as ammonia monooxygenase (AMO), nitrite oxidoreductase (NOR), nitrate reductase (NAR), nitrite reductase (NIR), exopolyphosphatase (PPX), and polyphosphate kinase (PPK), which are associated with the nitrogen and phosphorus removal processes, were investigated. Finally, the potential effects of MWCNTs on bacterial communities in activated sludge were studied by MiSeq sequencing.

## Materials and Methods

### 2.1 Preparation of MWCNTs

The MWCNTs used in this study were purchased from a commercial company (Shenzhen Nanotech Co., Shenzhen, China). According to the manufacturers report, the sample is over 97% (by mass) MWCNTs, with a powder-specific surface area between 40 and 70 m^2^/g. The MWCNTs have an average diameter of 40–60 nm and an average length of 5–15 µm. The impurities of these commercial MWCNTs are less than 3% (by wt). A 100 mg/L MWCNTs stock solution was prepared for this study by adding 100 mg MWCNTs to 1.0 L Milli-Q (Molsheim, France) water, followed by 1 h of ultrasonication (25°C, 120 W, 40 kHz). In this study, 1 mg/L was chosen as the environmentally relevant concentration of MWCNTs, and 20 mg/L was also investigated because WWTPs may treat high concentrations of MWCNTs in wastewater released from MWCNTs manufacturing plants.

### 2.2 Activated sludge cultures and sampling

Three groups of sequencing batch reactors (SBRs) were operated in this study (SBR1, SBR2 and SBR3), and each group was set up in triplicate. Each SBR has a 4-L working volume, with 1 L of inoculated sludge obtained from a municipal WWTP in Beijing, China. After dividing seed activated sludge into the three groups of SBRs, the three groups of reactors were operated for more than 40 days to achieve a stable nitrogen and phosphorus removal performance, approximately 83% and 95% respectively. The operating temperature was maintained at (21±3) °C, and operated on three 8 h cycles every day. Each cycle consisted of a 1.5-h anaerobic and a 3-h aeration period, followed by 1.5 h of settling, then 10 min of decanting, and finally, a 110-min idle period. After the settling phase, 2 L of the supernatant was discharged from each SBR, and was replaced with 2 L of 0 (SBR1), 1 (SBR2), and 20 mg/L (SBR3) MWCNTs wastewater, respectively, during the initial 10 min of a new cycle (the anaerobic period). The hydraulic retention time in these three SBRs was 16 h, and the sludge retention time was maintained at approximately 20 days by sludge discharging. During the aeration period, the air was controlled by an air flow meter to maintain the DO in the SBRs between 0.9 and 2.5 mg/L. The initial concentrations of COD, ammonia-nitrogen (NH_4_^+^-N), and total phosphorus (TP) in the influent synthetic wastewater were maintained at approximately 500, 30, and 5 mg/L, respectively. The influent pH was maintained between 7.2 and 7.6.

### 2.3 Activated sludge respiration inhibition test

Five concentrations of MWCNTs in Milli-Q water were prepared according to the description given in 2.1. The final concentrations for the 500-mL volume of each reactor were 0.104, 0.502, 1.50, 2.36, and 3.20 g/L; these certain concentrations were based on relevant research by Luongo LA et al. [23]. Activated sludge was obtained from the aeration tank of the WWTP. After the concentrations of mixed liquor suspended solids (MLSS) were measured, the mixed liquor was washed by distilled water three times. The mixed liquor was concentrated to a MLSS of (4000±200) mg/L based on the initial MLSS information. A series of samples were prepared by combining the pre-concentrated activated sludge with synthetic wastewater (16 g peptone, 11 g meat extract, 3 g urea, 0.7 g NaCl, 0.4 g CaCl_2_·2H_2_O, 0.2 g MgSO_4_·7H_2_O, and 2.8 g K_2_HPO_4_ and the chemicals were mixed in 1 L of distilled water), a reference substance (3, 5-dichlorophenol) or MWCNTs, and distilled water (Table S1 in File S1). Each reactor had a final volume of 500 mL, and the operation temperature was (20±2) °C. During the aeration contact, each reactor was aerated with Oil-less Vacuum Pump (Qingdao, China) at a fixed air flow of 1 L/min. At the end of the aeration contact, the content of each reactor was transferred to a 500-mL jar with a gasket seal to guarantee sealability between the jar and the DO meters. Then, the DO readings were recorded at 1-min intervals for 10 min and the unit of the respiration rate is mg O_2_/L/h. The percent inhibition was obtained from Eq.1, which was based on the average respiration rate.
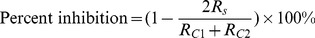
(1)


Where Rs is respiration rate from MWCNTs (test substance), mg O_2_/L/h; R_C1_ is control 1 respiration rate, obtained at the beginning of the test, mg O_2_/L/h; R_C2_ is control 2 respiration rate, obtained at the end of the test, mg O_2_/L/h.

### 2.4 Short-term and long-term exposure to MWCNTs

To measure the effects of short-term exposure, data were obtained after three operation cycles (1 day) at MWCNT influent concentrations of 1 and 20 mg/L. After three cycles, the activated sludge in the SBRs was washed three times with a 0.9% NaCl solution before feed wastewater without MWCNTs to achieve the same influent component in each SBR [12]. During the fourth cycle, the changes of NH_4_^+^-N, nitrate-nitrogen (NO_3_^−^-N), nitrite nitrogen (NO_2_^−^-N), TP, sludge viability, Sludge Volume Index (SVI), and the activities of AMO, NOR, NAR, NIR, PPX, and PPK were measured. After the short-term exposure test, all the SBRs were continuously operated to investigate the chronic effects of MWCNTs by feeding wastewater contained different concentrations of MWCNTs as mentioned in 2.2. During the long-term exposure study, the effluent concentrations of COD, NH_4_^+^-N, NO_3_^−^-N, NO_2_^−^-N, and TP were measured and recorded every 3 days. After 180 days, effluent concentrations of COD, NH_4_^+^-N, NO_3_^−^-N, NO_2_^−^-N, and TP were relatively stable, and changes of NH_4_^+^-N, NO_3_^−^-N, NO_2_^−^-N, and TP, sludge viability, SVI, and the activities of AMO, NOR, NAR, NIR, PPX, and PPK during one cycle were measured again.

The analyses of NH_4_^+^-N, NO_3_^−^-N, NO_2_^−^-N, TP, MLSS, and MLVSS are detailed in the Standard Methods [24]. The measurements of the activities of AMO, NOR, NR, NIR, PPX, and PPK were based on the methods of Zheng, et al. and Chen, et al. [12],[25].

### 2.5 DNA extraction

In the final week of the study, nine activated sludge samples were collected from SBR1 (A1, A2, and A3), SBR2 (B1, B2, and B3), and SBR3 (C1, C2, and C3) at the aerobic stage, and each sample was dispensed into a 1.5 mL sterile Eppendorf tube and centrifuged at 14,000×*g* for 10 min. The supernatant was decanted, and the pellets were stored at −20°C prior to analysis.

The pellets of activated sludge samples were washed three times by centrifugation using sterile high-purity water for 5 min at 15,000×*g*. DNA extraction was then performed using a FastDNA SPIN Kit for Soil (MP Biotechnology, Illkirch, France) according to the manufacturers protocol. Before sequencing, the extracted DNA samples were amplified with a set of primers targeting the hypervariable V4 region of the 16S rRNA gene. The forward primer is 5′-GTGCCAGCMGCCGCGG-3′ and the reverse primer is 5′-GGACTACHVGGGTWT CTAAT-3′. Barcode and adapter were incorporated between the adapter and the forward primers. The PCR amplification was conducted in a 20-µL reaction system containing 4 µL 5×FastPfu Buffer, 2 µL 2.5 mM dNTPs, 0.4 µL Forward Primer (5 µM), 0.4 µL Reverse Primer (5 µM), 0.4 µL Fastfu Polymerase, and 10 ng Template DNA. The PCR was performed under the following conditions: 95°C for 2 min; 30 cycles of 95°C for 30 s, 55°C for 30 s, 72°C for 30 s, and a final extension at 72°C for 5 min. PCR products were purified using QIAquick PCR Purification Kit (Qiagen, Germany).

### 2.6 MiSeq sequencing

About 500 ng of purified PCR product for each sample was mixed and sent to a commercial company (Majorbio, Shanghai, China) for Illumina MiSeq sequencing. After sequencing, Python scripts were written to perform quality filtering of the raw reads as follows: (1) to sort sequences exactly matching the specific barcodes into different samples, (2) to check sequencing quality by filtering out the reads with any uncalled bases or two-paired end reads with less than 80 bases overlapping, (3) to trim off the barcodes and primers. Taxonomic classifications of the effective sequences were carried out using the RDP Classifier (Version 2.4) with a set confidence threshold of 80% to assign the sequences to different taxonomy levels. The raw reads have been deposited into the NCBI short-reads archive database (Accession Number: SRR1174196).

### 2.7 Statistical analysis

The Shannon-Wiener index was calculated to assess bacterial diversity. Detrended correspondence analysis (DCA) was performed to examine the overall variation among bacterial communities of these nine samples. Partial Mantel test was used to reveal relationships between bacterial community dynamics and operational and environmental parameters. All the statistical analysis were performed using the VEGAN package in R (v.2.15.1; http://www.r-project.org/).

All tests were conducted in triplicate, and an analysis of variance (ANOVA) was used to test the significance of results and *p*0.05 was considered to be statistically significant.

## Results and Discussion

### 3.1 Effects of MWCNTs on nutrient removal from wastewater

**Figure 1:**
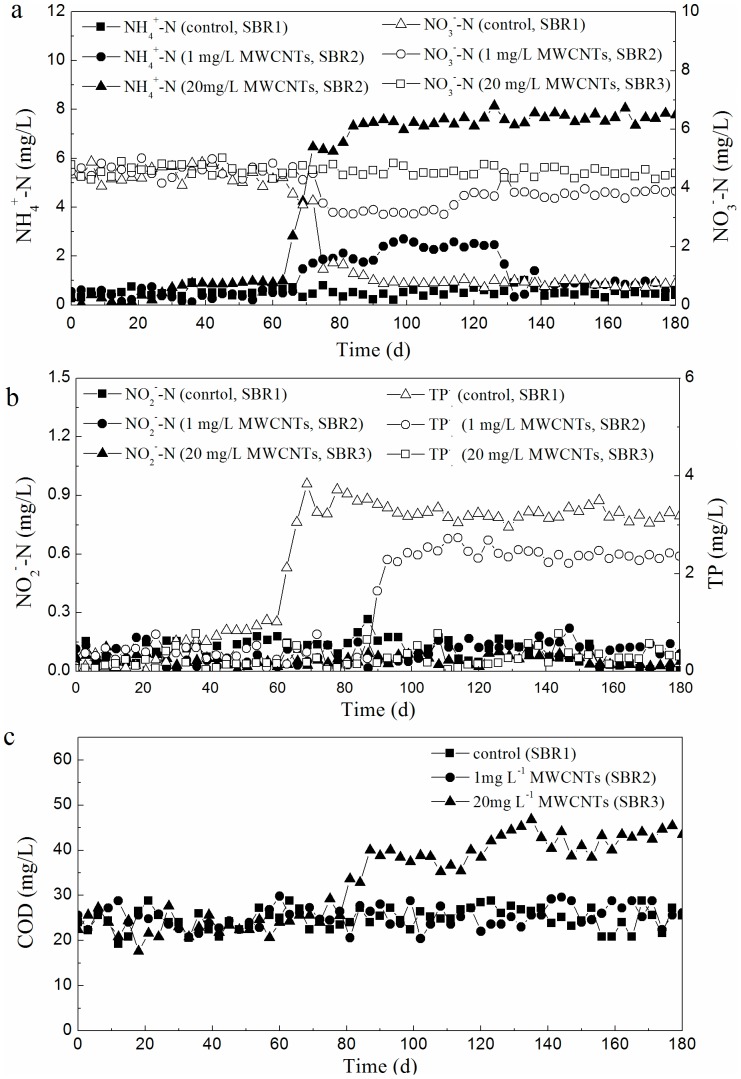
Variations of effluent concentrations of NH_4_^+^-N, NO_3_^−^-N, NO_2_^−^-N, TP, and COD under exposure to different concentrations of MWCNTs. All standard deviations of triplicate measurements are less than 20%.

As shown in Fig. 1b and 1c, the effluent COD and NO_2_^−^-N concentrations in the presence of 1 mg/L MWCNTs (SBR2) were relatively stable with increasing exposure time, and similar to those in the control SBR (SBR1) over a period of 180 days. However, the effluent concentration of NH_4_^+^-N (Fig. 1a) showed a clear increasing trend after 70 days, and stabilized at approximately 2.5 mg/L. The effluent NO_3_^−^-N concentration decreased from 4.4 to 3.3 mg/L due to the deterioration of ammonia oxidation; following this, the effluent NH_4_^+^-N concentration decreased to a relatively low level (0.86 mg/L) after a period of 30 days, and the concentration of NO_3_^−^-N increased and stabilized around 3.9 mg/L. In the treatment containing 1 mg/L MWCNTs, there was a period of adverse effects on ammonia oxidation process during the long-term exposure, however, the concentrations of NH_4_^+^-N and NO_3_^−^-N returned to normal after 60 days. The average total nitrogen (TN) removal efficiency in SBR2 did not change significantly and remained around 83.8%. By contrast, in the activated sludge exposed to 20 mg/L MWCNTs in SBR3, there was also a decrease in the oxidation of effluent NH_4_^+^-N, and the NH_4_^+^-N effluent concentration increased significantly from 0.3 to approximately 7.5 mg/L and did not decrease back to normal concentrations until the end of the study. After 180 days of exposure, the effluent NO_3_^−^-N and NO_2_^−^-N concentrations in SBR3 were around 0.76 and 0.16 mg/L, respectively. The average TN removal efficiency of SBR3 declined from 84.0% to 71.9%. The phosphorus removal efficiencies were both significantly influenced over the course of long-term exposure of 1 and 20 mg/L MWCNTs. The effluent TP in SBR2 and SBR3 increased to 3.3 and 2.4 mg/L over a period of 80 and 100 days, respectively. A MWCNT concentration of 20 mg/L adversely impacted COD removal to some extent, and the concentration of effluent COD was statistically higher (42.8 mg/L) than that in SBR1 (24.0 mg/L) (*p*0.05) and SBR2 (25.0 mg/L) (*p*0.05). This result was different from those of some researchers [21],[23] who concluded that CNTs can absorb soluble COD because of their large surface area to volume ratio. These studies were focused on the shock loaded, or short-term effects (less than 1 month) of CNTs, but the long-term effects of 20 mg/L MWCNTs in this study showed inhibitory effects on COD removal; this might be because the long-term accumulation of MWCNTs appears to lead to an increase in antimicrobial properties. 20 mg/L MWCNTs did adversely impact nutrient and COD removal in activated sludge process over the long term. However, 1 mg/L MWCNTs only influenced phosphorus removal in activated sludge process, and the adverse effect emerged later than in the 20 mg/L MWCNT treatment.

**Figure 2:**
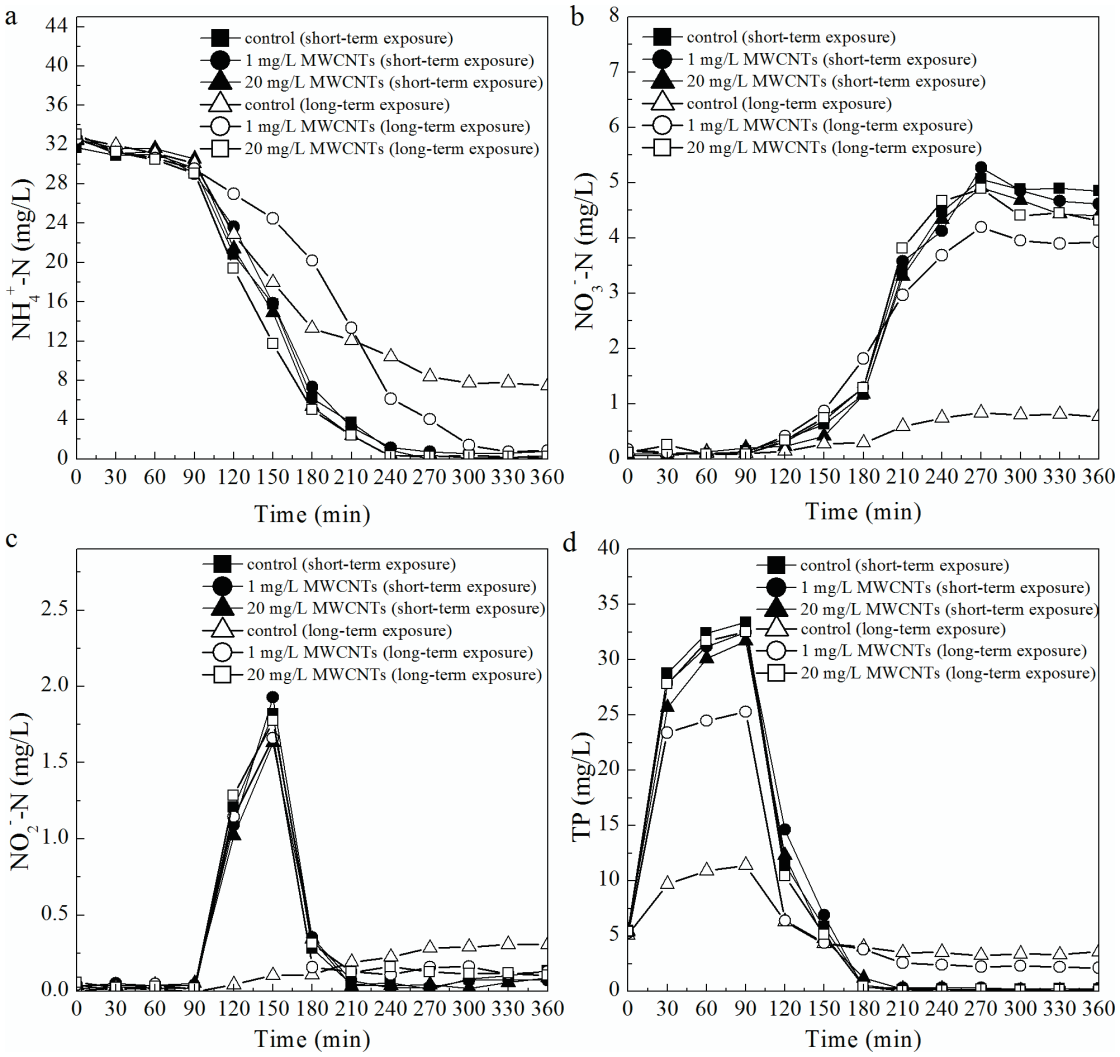
Variations of NH_4_^+^-N, NO_3_^−^-N, NO_2_^−^-N, and TP during one cycle after short-term and long-term exposure to different concentration of MWCNTs were investigated. All standard deviations of triplicate measurements are less than 20%.

Changes of NH_4_^+^-N (Fig.2, a), NO_3_^−^-N (Fig. 2, b), NO_2_^−^-N (Fig. 2, c), and TP (Fig. 2, d) concentrations were observed during one cycle after the short-term and long-term exposure of different concentrations of MWCNTs. The changes of NH_4_^+^-N, NO_3_^−^-N, NO_2_^−^-N, and TP after short-term exposure of MWCNTs did not differ significantly (*p*0.05) between SBR1, SBR2, and SBR3; it was also reported that MWNTs did not show any significant antimicrobial activity at concentrations up to 500–875 mg/L after short-term exposure [18]. After long-term (180 days) exposure, the variations of NH_4_^+^-N, NO_3_^−^-N, and TP in SBR2 and SBR3 changed significantly. Although the effluent concentration of NH_4_^+^-N did not differ significantly between SBR1 and SBR2, the variations of NH_4_^+^-N differed between the two SBRs, especially during the aeration stage (Fig. 2, a), and the occurrence of the minimum NH_4_^+^-N concentration was delayed and appeared an hour later than that in SBR1. Changes in the concentration of NH_4_^+^-N in SBR3 declined much slower during the aeration stage (after 90 min) than that in SBR1, and stabilized around 7.5 mg/L, which was higher than that in SBR1 (0.3 mg/L). Following a deterioration of ammonia oxidation process, the effluent NO_3_^−^-N concentration decreased from 4.5 to 0.76 mg/L and the average TN removal efficiency in SBR3 decreased from 84.0 to 71.9%. Furthermore, changes in anaerobic phosphorus release, aerobic phosphorus uptake, and phosphorus removal were influenced in varying degrees by the presence of different concentrations of MWCNTs. Long-term exposure to 1 and 20 mg/L MWCNTs induced both poor phosphorus release and phosphorus uptake, and these inhibitory effects were more serious in the presence of 20 mg/L MWCNTs than 1 mg/L of MWCNTs. The average TP removal efficiencies in SBR2 and SBR3 were decreased from 96.8% to 52.3% and from 98.2% to 34.0% respectively, which were much lower than that in SBR1 (99.8%).

### 3.2 Respiration inhibition test

Results of the respiration inhibitions tests at different MWCNTs concentrations are illustrated in Table S2 in File S1. The average respiration rate of the control group was (24±1.9) mg O_2_/L/h. MWCNTs had no significant respiration inhibitory effects when concentrations were less than 0.5 g/L, however, at concentrations of MWCNTs above 1 g/L level, inhibitory effects are observed. For example, at 1.496 g/L MWCNTs, respiration inhibition was (27.5±7.5)%. It is obvious that respiration inhibition progressively increases as the MWCNTs concentration increases, and the highest concentration of MWCNTs in this study (3.196 g/L) resulted in the highest respiration inhibition ((62.5±5.5)%), these results were similar to the major conclusion of Luongo LAs study. These results could explain the finding that short-term exposure of MWCNTs had no significant influence on activated sludge process, but long-term exposure of different concentrations of MWCNTs resulted in accumulation of MWCNTs leading to high concentrations in both SBR2 and SBR3, and these concentrations resulted in negative effects on activated sludge processes.

### 3.3 Long-term effects of MWCNTs on the activity of key enzymes

**Figure 3:**
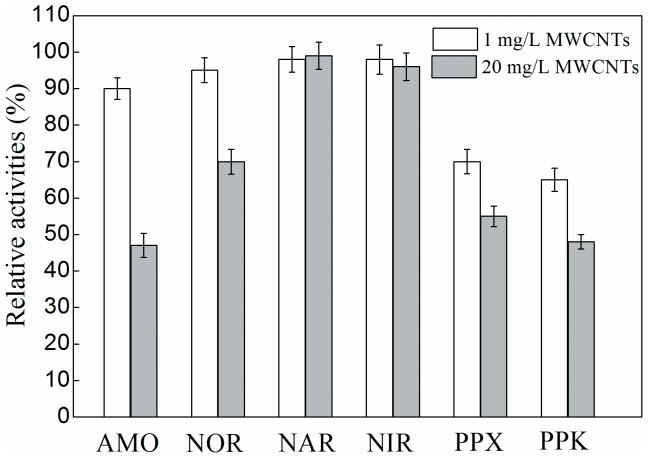
Long-term effects of different concentrations of MWCNTs on activities of key enzymes, the activities of key enzymes were presented as relative activities compared with control activated sludge (% of control). Error bars represent standard deviation of three replicates.

Six key enzymes of activated sludge were investigated after long-term effects of different concentrations of MWCNTs (Fig. 3). The results demonstrated that the relative activities of AMO and NOR significantly decreased (*p*0.05) after long-term exposure to 20 mg/L MWCNTs. AMO and NOR are two key enzymes in the process of nitrification [15], therefore these observations explain the observed high effluent NH_4_^+^-N concentrations and low TN removal efficiencies after long-term exposure to 20 mg/L MWCNTs. However, there was no observed impact of long-term exposure to 1 and 20 mg/L MWCNTs on the activity of NAR and NIR. Furthermore, long-term exposure to 1 and 20 mg/L MWCNTs caused a decrease in the relative activities of PPX and PPK (*p*0.05), which directly relate to phosphorus removal, and explain the lower phosphorus release and uptake in the presence of different concentrations of MWCNTs. Additionally, these results also indicated that higher concentration of MWCNTs had more inhibitive effects on the activities of PPX and PPK (Fig. 3).

### 3.4 Shifts in the bacterial community structure in activated sludge after long-term exposure to MWCNTs

As shown in Table 1, after filtering the low-quality reads, there were 23,305–43,910 effective reads for nine activated sludge samples; the library size of each sample was then normalized to 23,305 sequences, which was the lowest number of sequences among the nine samples. Ribosomal Database Project (RDP) Classifier was used to assign these sequence tags into different operational taxonomic units (OTU) with a 3% of nucleotide cutoff. A total of 1142 OTUs were obtained from nine activated sludge samples. The average OTUs of the samples in SBR1 was 817, however, the average OTUs in the presence of 1 and 20 mg/L MWCNTs were 644 and 532, respectively. It is clear that long-term exposure of these two concentrations MWCNTs significantly decreased the amount of OTUs in activated sludge. The average Shannon-Wiener index in SBR1 (5.18) was significantly higher (*p*0.05) than that in the presence of 20 mg/L MWCNTs (SBR3, averaging 3.72), and the inhibitory effect of long-term exposure to 20 mg/L MWCNTs was more severe than 1 mg/L MWCNTs (SBR2, averaging 4.47). Long-term exposure to 20 mg/L MWCNTs clearly caused a reduction in the bacterial diversity in activated sludge.

**Table 1:** Bacterial diversity indices of nine activated sludge samples from three sequencing batch reactors (3% cutoff).

samples	sequences	OTUs	Shannon-Wiener
A1	33680	822	5.14
A2	23305	816	5.06
A3	39737	815	5.35
B1	40746	647	4.47
B2	47805	679	4.54
B3	29748	607	4.41
C1	42452	509	3.50
C2	27659	561	3.74
C3	43910	526	3.91

**Figure 4:**
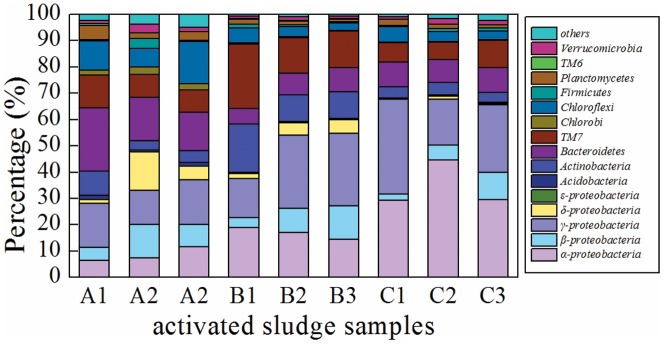
Abundances of different phyla and classes in *Proteobacteria* in the nine activated sludge samples. The abundance is presented in terms of percentage in total effective bacterial sequences in a sample.

As shown in Fig. 4, *Proteobacteria* was the predominant phylum in all samples, accounting for 29.4%–69.0% of total effective bacterial sequences. Notably, the abundance of *Proteobacteria* in all activated sludge samples of SBR3 (C1 68.1%, C2 69.0%, and C3 66.2%) were much higher (*p*0.05) than that in SBR1 (A1 29.4%, A2 47.6%, and A3 42.4%). It is obvious that long-term exposure to MWCNTs have increased the abundance of *Proteobacteria*. Other dominant phyla were *Actinobacteria* (3.4%–18.2%), *Bacteroidetes* (5.9%–23.9%), *Chloroflexi* (2.6%–15.9%), and *TM7* (6.6%–24.5%). Besides *Proteobacteria*, the abundances of these dominant phyla were also different between the three reactor groups. For example, *Bacteroidetes* and *Chloroflexi* in SBR1 composed a greater proportion of the total effective bacterial sequences than that in SBR2 and SBR3.

Within *Proteobacteria*, γ*-proteobacteria* was the most dominant class in the activated sludge samples of SBR1, and the abundance of α- and β*-proteobacteria* composed a similar proportion of SBR1 samples. The most dominant class within *Proteobacteria* in SBR2 was also γ*-proteobacteria*, but the abundance of α*-proteobacteria* was higher than β*-proteobacteria* in this group. However, α*-proteobacteria* was the dominant class in activated sludge samples from SBR3. Compared with SBR1, MWCNTs increased the abundance of α*-proteobacteria* and γ*-proteobacteria*, and finally induced the increase of the abundance of *Proteobacteria* in SBR2 and SBR3.

The abundance of α*-proteobacteria* in activated sludge samples showed an increase trend between the three groups. Compared with the control activated sludge samples (A1, A2, and A3), after long-term exposure to MWCNTs, the proportion of α*-proteobacteria* in SBR2 samples (averaging 16.7%) and SBR3 samples (averaging 34.4%) was significantly higher (*p*0.05) than that in control samples (averaging 8.5%). ε-*Proteobacteria* composed a very low proportion (0%–0.17%) of the three groups of samples, and the ε-subdivision did not appear at all in the control sludge samples.

**Figure 5:**
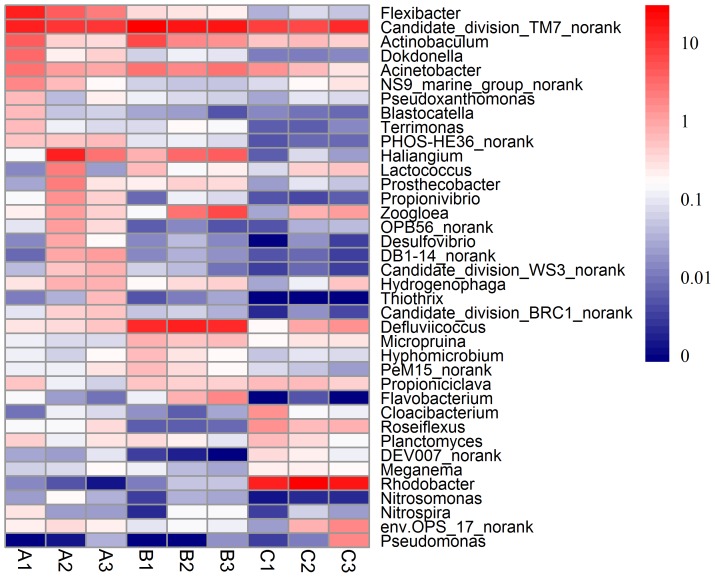
Heat map of top 10 genera in each sample. Total 36 genera were selected from nine samples, and the color intensity in each panel shows the percentage of a genus in a sample, referring to color key at the right side.

282 genera were classified in the 9 activated sludge samples; among them, 102 genera were shared by all nine samples, and 173 genera were identified in at least six samples (Table S3 in File S1). A total of 33 rare genera, which were only identified in one or two samples, were identified. Fig. 5 shows the top 10 genera in each sample, and the abundance of a total of 38 genera are compared in this figure. Most of the genera occur in relative high abundance (about 25 genera, composing a proportion greater than 0.3%) in the SBR1 samples. In addition, the richness and evenness of genera in the SBR1 samples were greater than those in SBR2 and SBR3. By contrast, most genera (about 20) composed a low proportion (less than 0.3%) in the activated sludge samples after long-term exposure to 20 mg/L MWCNTs. The abundance of *Thiothrix* in SBR2 samples was much lower (*p*0.05) than that of SBR1, and *Thiothrix* was not present at all in SBR3 samples. This genus was widely observed in WWTPs, and usually contributes to the problem of filamentous sludge bulking [26]. this might demonstrate that MWCNTs can improved sludge settle ability by decreasing the abundance of certain bacteria which may induce sludge bulking. This result might support the conclusion of Yins [20] that SWCNTs can improve sludge settle ability. Furthermore, MWCNTs influenced the abundances of nitrifiying bacteria and polyphosphate-accumulating organisms (PAOs). In the activated sludge of SBR1, *Nitrosomonas* and *Nitrospira* are two typical genera of ammonia-oxidizing bacteria (AOB) and nitrite-oxidizing respectively (NOB) (Fig. 5), and AOB and NOB were mainly responsible for the oxidation of ammonia to nitrate [12]. 1 and 20 mg/L MWCNTs obviously reduced the abundance of *Nitrosomonas*, which might explain the deterioration of the ammonia oxidation process in the presence of 1 and 20 mg/L MWCNTs. It should be noted that 20 mg/L MWCNTs deceased the abundance of *Nitrospira*, and these might be the main reason for the changes of NO_3_^−^-N and NO_2_^−^-N in SBR3. By contrast, the average abundance of *Nitrospira* (0.3%) in the activated sludge of SBR2 was similar to that in SBR1 (0.4%) after long-term exposure to 1 mg/L MWCNTs. *Rhodobacter* composed a high proportion (12.5%–25.6%) in SBR3 samples, and species in the genus *Rhodobacter* are known to have denitrifying function, this might explain that denitrifying process was not inhibited after long-term exposure to 20 mg/L MWCNTs. Regarding of PAOs, *Acinetobacter* and *Pseudomonas* were detected in activated sludge, which were usually reported to carry out wastewater phosphorus removal. The results indicated that the average abundance of PAOs (*Acinetobacter* and *Pseudomonas*) in SBR3 accounted for 1.7% of total biomass after long-term exposure to 20 mg/L MWCNTs, which was less than that in SBR1 (averaging 2.7%). This might be the main reason for the deterioration of wastewater phosphorus removal in SBR3. Interestingly, the abundance of PAOs (averaging 2.5%) in SBR2 was similar to that in control SBR after long-term exposure to 1 mg/L MWCNTs. But, the abundances of *Defluviicoccus* (9.4%–11.2%) and *Micropruina* (1.1%–1.4%) were much higher than those in SBR1 samples. *Defluviicoccus and Micropruina* are thought to be glycogen-accumulating organisms (GAOs) which inhibited the phosphorus removal in activated sludge process by competing with PAOs [27],[28]. The increased abundances of GAOs may be one of the reasons of deterioration of phosphorus removal.

**Figure 6:**
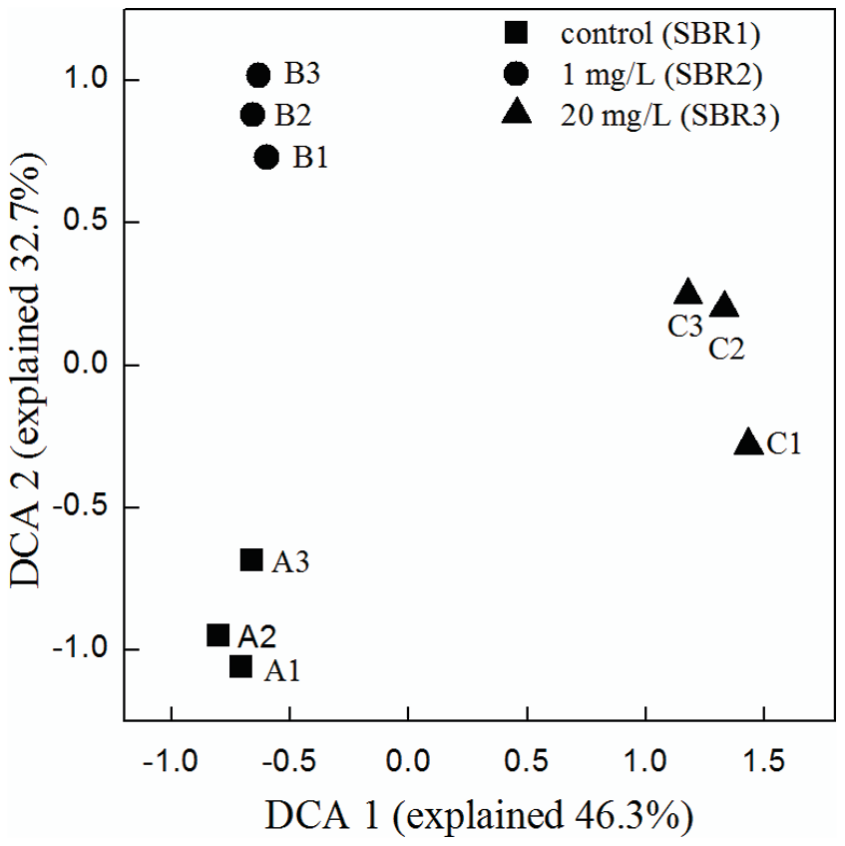
Detrended correspondence analysis (DCA) of sequencing data in nine samples.

DCA of sequencing data was performed to examine the overall variation within and between bacterial communities of these nine activated sludge samples (Fig. 6). DCA is an ordination technique that uses detrending to remove the arch effect typical in correspondence analysis [29]. The results illustrated that samples collected from the same SBR group were gathered together. However, bacterial community structures of activated sludge in the presence of 1 (B1, B2, and B3) and 20 mg/L (C1, C2, and C3) MWCNTs clustered far away from the cluster of control samples (A1, A2, and A3).

Partial Mantel tests (Table 2) were carried out to investigate the correlation between bacterial community structure and operation, and environmental parameters (MWCNTs, pH, DO, temperature, COD, NH_4_^+^-N, and TP, Table S4 in File S1). The results showed that MWCNTs (*p*0.01) and DO (*p*0.05) were positively correlated with bacterial community structure. DO is well recognized as a critical process parameter in biological wastewater treatment processes. Park et al. [30] have found that DO can influence bacterial community structure, and the result of this study also supports that conclusion. The presence of MWCNTs was the most important factor shaping bacterial community structure, and the cytotoxicity of MWCNTs might contribute to that close relationship between bacterial community structure and the presence of MWCNTs. In addition to those deterministic factors (MWCNTs, wastewater, and operational variables) as mentioned above, the stochastic factors (random immigration and births/deaths) may also affect the structure of the bacterial community. By studying four activated sludge bioreactors, Ayarza and Erijman [31] demonstrated that both neutral and deterministic effects operated simultaneously in the assembly of bacterial floc. Although the immigrating rates of synthetic wastewater used in this study might be less than those of municipal wastewater [32], the effects other stochastic factors on microbial communities warrants future investigation. To date, when it comes to mechanisms of cytotoxicity induced by CNTs, there are several potential mechanisms. (1) CNTs may induce the generation of reactive oxygen species [33],[34]; (2) CNTs may destroy the bacterial cell [35]; and (3) certain impurities of CNTs and chemical modifications may be associated with their cytotoxicity. To date, the second mechanism has obtained more support than the other two. Kang et al. [17] found that CNTs direct contact with the cell membrane cause damage in the cell membrane, and finally induce DNA and RNA efflux from microbial cells. These mechanisms of the toxicity imposed by CNTs might explain the significant correlation between MWCNTs and bacterial community structure.

**Table 2:** Relationship between operation parameters and bacterial community structure in nine samples.

Operation parameters	*r_M_* a	P value
MWCNTs	0.881	0.00300
pH	0.0712	0.344
DO	0.473	0.034
T	0.155	0.128
COD	0.0508	0.346
NH_4_^+^-N	−0.0185	0.455
TP	0.0728	0.304

a *r_M_*, Mantels correlation coefficient.

## Supporting Information

File S1 Contains the following files: **Table S1**. Contents of each reactor utilized in respiration inhibition test. **Table S2**. Respiration rate of activated sludge. **Table S3**. Shared genera among different numbers of samples. **Table S4**. Operation conditions of each SBR when activated sludge samples were collected.
(DOCX)
