# Supplementary material for: Impacts of Multiwalled Carbon Nanotubes on Nutrient Removal from Wastewater and Bacterial Community Structure in Activated Sludge
Source: PLoS One. 2014 Sep 19;9(9):e107345. doi: 10.1371/journal.pone.0107345 (PMC4169552; doi:10.1371/journal.pone.0107345)
Supplement: File S1 — Contains the following files: Table S1. Contents of each reactor utilized in respiration inhibition test. Table S2. Respiration rate of activated sludge. Table S3. Shared genera among different numbers of samples. Table S4. Operation conditions of each SBR when activated sludge samples were collected. (DOCX) [file pone.0107345.s001.docx]

**Supporting Information Legends**

Table S1 Contents of each reactor utilized in respiration inhibition test

Table S2 Respiration rate of activated sludge

Table S3 Shared genera among different numbers of samples

Table S4 Operation conditions of each SBR when activated sludge samples were collectedTable S1 Contents of each reactor utilized in respiration inhibition test

|  | Activated sludge (mL) | Synthetic wastewater (mL) | DI (mL) | Volume used (mL) | | Final concentration | |
| --- | --- | --- | --- | --- | --- | --- | --- |
| Control 1 | 200 | 16 | 284 | 0 | 0 | |  |
| MWCNTs | 200 | 16 | 184 | 100 (519mg/L) | 0.104g/L | |  |
|  | 200 | 16 | 184 | 100 (2.51mg/L) | 0.502g/L | |  |
|  | 200 | 16 | 184 | 100 (7.48mg/L) | 1.50g/L | |  |
|  | 200 | 16 | 184 | 100 (11.85mg/L) | 2.36g/L | |  |
|  | 200 | 16 | 184 | 100 (15.98g/L) | 3.20g/L | |  |
| 3,5-dichlorophenol (0.5g/L) | 200 | 16 | 281.5 | 2.5 | 2.5mg/L | |  |
|  | 200 | 16 | 279 | 5 | 5mg/L | |  |
|  | 200 | 16 | 274 | 10 | 10mg/L | |  |
|  | 200 | 16 | 259 | 25 | 25mg/L | |  |
| Control 2 | 200 | 16 | 284 | 0 | 0 | |  |

Table S2 Respiration rate of activated sludge

| Concentration of MWCNTs (g L^-1^) | Respiration rate（mgO_2_ L^-1^ h^-1^)） | Respiration inhibition（%） |
| --- | --- | --- |
| Control average | 24±1.9 | 0 |
| 0.104 | 22.8±1.2 | 5±5 |
| 0.502 | 21.0±0.9 | 12.5±3.75 |
| 1.496 | 17.4±1.8 | 27.5±7.5 |
| 2.360 | 13.8±0.6 | 42.5±2.5 |
| 3.196 | 9.0±1.2 | 62.5±5.5 |

Table S3 Shared genera among different numbers of samples

| Number of sample | Number of shared genera | Percentage in classified genera |
| --- | --- | --- |
| 9 | 102 | 37.0 |
| 8 | 130 | 47.1 |
| 7 | 156 | 56.5 |
| 6 | 173 | 62.6 |
| 5 | 198 | 71.7 |
| 4 | 226 | 81.9 |
| 3 | 243 | 88.0 |
| 2 | 261 | 94.6 |
| 1 | 276 | 100.0 |

Table S4 Operation conditions of each SBR when activated sludge samples were collected.

| Characteristics | A1 | A2 | A3 | B1 | B2 | B3 | C1 | C2 | C3 |
| --- | --- | --- | --- | --- | --- | --- | --- | --- | --- |
| MWCNTs concentration(mg/L) | 0 | 0 | 0 | 1 | 1 | 1 | 20 | 20 | 20 |
| pH | 7.5 | 7.4 | 7.6 | 7.5 | 7.2 | 7.4 | 7.3 | 7.5 | 7.6 |
| Dissolved oxygen (mg/L) | 2.3 | 2.6 | 2.5 | 2.5 | 2.4 | 2.1 | 2.6 | 2.7 | 2.5 |
| Mixed liquor temperature (^o^C) | 26.1 | 24.5 | 25.5 | 24.5 | 21 | 21.5 | 25.5 | 20.7 | 21.6 |
| Influent COD (mg/L) | 517.9 | 520.4 | 560.3 | 549.8 | 560.5 | 530.4 | 548.4 | 501.8 | 540.5 |
| Influent NH_4_^+^-N (mg/L) | 35.2 | 36.4 | 30.5 | 36.8 | 32.2 | 30.7 | 33.2 | 30.2 | 31.9 |
| Influent TP (mg/L) | 5.4 | 5.2 | 4.8 | 5.8 | 5.2 | 4.9 | 5.6 | 4.9 | 5.3 |

The characteristic values of operation conditions were average values of one week before these activated sludge samples were collected.
